# Association Between the Inflammatory Biomarkers and Left Ventricular Systolic Dysfunction in Patients with Exacerbations of Chronic Obstructive Pulmonary Disease

**DOI:** 10.4274/balkanmedj.2016.1114

**Published:** 2017-05-15

**Authors:** Ljiljana Andrijevic, Senka Milutinov, Ilija Andrijevic, Daniela Jokic, Marija Vukoja

**Affiliations:** 1 University of Novi Sad, Faculty of Medicine, Novi Sad, Serbia; 2 Oncology Institute of Vojvodina, Sremska Kamenica, Serbia; 3 The Institute for Pulmonary Diseases of Vojvodina, Sremska Kamenica, Serbia; 4 Zvezdara Health Center, Belgrade, Serbia

**Keywords:** Chronic obstructive pulmonary disease, inflammatory biomarkers, heart failure

## Abstract

**Background:**

Cardiovascular diseases are an important cause of morbidity and mortality in chronic obstructive pulmonary disease patients. The increased inflammatory biomarker levels predict exacerbations and are associated with cardiovascular diseases in stable chronic obstructive pulmonary disease patients but their role in the settings of acute chronic obstructive pulmonary disease exacerbations has not been determined.

**Aims:**

To analyse the association between inflammatory biomarkers and heart failure and also to determine the predictors of mortality in patients with exacerbations of chronic obstructive pulmonary disease.

**Study Design:**

Prospective observational study.

**Methods:**

We analysed 194 patients admitted for acute exacerbation of chronic obstructive pulmonary disease at The Institute for Pulmonary Diseases of Vojvodina, Sremska Kamenica, Serbia. In all patients, C-reactive protein, fibrinogen, N-terminal of the pro-hormone brain natriuretic peptide and white blood count were measured and transthoracic echocardiography was performed.

**Results:**

There were 119 men (61.3%) and the median age was 69 years (interquartile range 62-74). Left ventricular systolic dysfunction (ejection fraction <50%) was present in 47 (24.2%) subjects. Patients with left ventricular systolic dysfunction had higher C-reactive protein levels (median 100 vs. 31 mg/L, p=0.001) and fibrinogen (median 5 vs. 4 g/L, p=<0.001) compared to those with preserved ejection fraction. The overall hospital mortality was 8.2% (16/178). The levels of C-reactive protein, fibrinogen, N-terminal pro-brain natriuretic peptide and ejection fraction predicted hospital mortality in univariate analysis. After adjusting for age, hypoxemia and C-reactive protein, ejection fraction remained significant predictors of hospital mortality (OR 3.89, 95% CI 1.05-15.8).

**Conclusion:**

Nearly a quarter of patients with the exacerbation of chronic obstructive pulmonary disease present with left ventricular systolic dysfunction which may be associated with mortality.

The exacerbation of chronic obstructive pulmonary disease (COPD) is an acute event, which disrupts the disease flow, and is defined as the deterioration of respiratory symptoms, worsening of dyspnoea and/or enhanced cough and/or the enhanced production of sputum. Exacerbation is accompanied by a change of the usual therapeutic treatments, mainly in terms of the introduction of antibiotics and/or systemic corticosteroids (1,2). Exacerbations of COPD are a major cause of hospitalisation and have a deleterious effect on life quality and survival. Prevention, recognition and adequate treatment of exacerbations are the most significant strategies in the choice of therapeutic approach to COPD (3,4). In recent decades, the concept of the occurrence of extra-pulmonary effects, and the development of comorbidities due to low level systemic inflammation in COPD is accepted. Therefore, the severity of COPD and further therapeutic treatment should be evaluated on the basis of a comprehensive and individualised approach to the patient and not just following the spirometric confirmation of the disease. Due to a better understanding of the pathogenic mechanisms and technological developments (tests are quick, relatively simple and cheap in regards to other diagnostic costs), the importance of the role of the various biomarkers in assessing the severity of disease and mortality increases rapidly. Elevated inflammatory biomarkers [C-reactive protein (CRP), fibrinogen and white blood cells (WBC)] in patients with COPD are related to increased exacerbations risk, even among patients with a mild form of COPD and those without previous exacerbations (5). High CRP, fibrinogen and WBC are linked to a double or to four-fold greater chance of comorbidities in stable COPD patients (6).

As cardiovascular diseases (CD) are among the most common causes of mortality in COPD, there is growing interest in the role of inflammatory and cardiac biomarkers in the assessment of disease severity and prediction of the mortality in COPD patients. The importance of inflammatory markers in stable COPD raises the question of how we can interpret these biomarkers during the COPD exacerbation in patients with cardiac insufficiency and how clinicians can use these biomarkers in the stratification of COPD comorbidities.

The purpose of our study was to analyse the association of inflammatory biomarkers and the presence of heart failure (HF) in patients hospitalised for acute exacerbation of COPD (AECOPD). Our secondary aim was to determine the significance of inflammatory biomarkers and HF as mortality predictors in AECOPD.

## MATERIALS AND METHODS

A prospective observational study of COPD patients who were consecutively admitted due to AECOPD at the Institute for Pulmonary Diseases of Vojvodina, Serbia, between July 2013 and September 2014. The ethics committee of the Institution approved the study and all patients signed the Informed consent form. All patients had severe forms of COPD (stage 3 and 4 according to GOLD), and met the criteria for a severe form of COPD exacerbation that required hospitalisation (1).

Demographic data were prospectively collected. Upon admission, serum levels of CRP, fibrinogen, N-terminal of the pro-hormone brain natriuretic peptide (NT-proBNP) and WBC count were measured. Transthoracic echocardiography (TTE) was performed on the GE Vivid 3 ultrasound system with a 1.7 MHz probe in all patients. The measurements were done in 2D and M-mode and using Doppler echocardiography. Assessment of the left ventricular ejection fraction (LVEF) was determined with Simpson's rule. We identified left ventricular systolic dysfunction (LVSD) based on a decrease in LVEF (<50%). In order to confirm the diagnosis and severity of HF, the NT-proBNP measurement was performed.

Based on echocardiographic assessment, all patients were classified into two groups:

1. COPD patients with normal LVEF,

2. COPD patients with observed LVSD.

### Statistical analysis

We compared baseline characteristics between COPD subjects with preserved LVEF and LVSD. Data are presented as median and interquartile range (IQR) and as whole numbers and percentages. The differences were analysed using the Mann-Whitney U test and Fisher's exact test as appropriate. Variables that influenced the mortality in univariate analysis (p<0.1) were entered into a multivariate stepwise regression model. The correlation between inflammatory markers and LVEF was analysed by Pearson coefficients. We considered p-values <0.05 to be statistically significant.

## RESULTS

The study included 194 patients, 119 men (61.3%), median age 69 years (IQR 62-74). All patients had been diagnosed with severe forms of COPD (stages 3 and 4) and none had pneumonia. The most common comorbidities were arterial hypertension (151/194, 77.8%), coronary artery disease (29/194, 14.9%) and diabetes (38/194, 19.6%). Chronic cor pulmonale was present in 34.4% (66/194) of patients.

Systolic dysfunction [ejection fraction (EF) <50%] was present in 47 (24.2%) patients. There was no difference in the frequency of LVSD between men and women. The median age of patients with LVSD was comparable to that of patients who had preserved EF (69 vs. 68 years, p=0.211). CRP (median 100 mg/L vs. 31 mg/L, p=0.001) and fibrinogen levels (median 5 g/L vs. 4 g/L, p<0.001) were higher in LVSD patients (Table 1).

There was a poor negative correlation between EF and CRP (r=-0.24, p<0.001) and no correlation between EF and fibrinogen (r=-0.04, p=0.543). The levels of NT-proBNP were moderately correlated with CRP (r=0.32, p<0.001), while poorly correlated with levels of fibrinogen (r=0.17, p=0.019).

**Figure 1:**
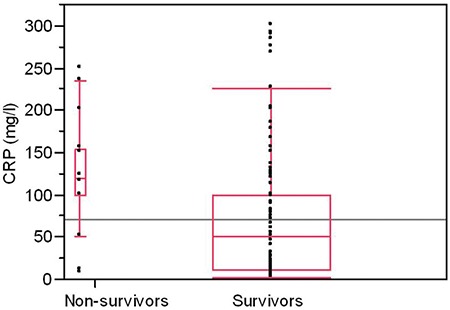
The association between C-reactive protein levels and hospital survival in acute exacerbation chronic obstructive pulmonary disease patients

**Figure 2:**
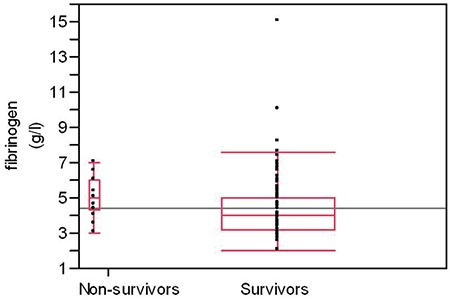
The association between fibrinogen levels and hospital survival in acute exacerbation chronic obstructive pulmonary disease patients

The overall hospital mortality was 8.2% (16/178). Hospital mortality was greater in COPD subjects who had EF <50% (19.2% vs. 4.8%, p=0.002) (Table 1). The CRP, fibrinogen, NT-proBNP, hypoxaemia and EF were linked to increased hospital mortality in univariate analysis (Table 2). We observed no association between the presence of comorbidities including chronic cor pulmonale and intra-hospital mortality. The relationship between markers of inflammation and survival is presented in Figure 1 and 2. In multivariate analysis, when adjusted for age, hypoxaemia and levels of CRP, EF <50% was independent predictor of hospital mortality (OR 3.89, 95% CI 1.05-15.8) (Table 3).

## DISCUSSION

This study demonstrated that there is a relatively high proportion of LVSD in hospitalised patients with AECOPD. The existence of LVSD was linked to greater levels of inflammatory biomarkers and increased hospital mortality.

In order to determine the severity of COPD exacerbations, there is a need for the fast stratification indicators (biomarkers or other tests) that would be used in the decision for the best hospital care unit choice for those patients (outpatient, hospital, hospital in intensive care unit) (7).

An inflammatory reaction in COPD involves an abnormal immune reaction and a constant activity of inflammatory mediators (cytokines, chemokines, and oxidising agents) which leads to structural changes in the bronchial system. Elevated levels of pro-inflammatory cytokines can be found in the sputum of COPD patients as well as high values of CRP, TNF-α and IL-6 in the plasma (8,9).

In our study, the median value of CRP in the group of patients with the exacerbation of COPD and concomitant systolic dysfunction (LVEF <50%) was 100 mg/L, and was higher than in the subjects without associated systolic dysfunction (average value of CRP was 31 mg/L). The significant difference was also observed in the levels of fibrinogen between the two groups.

The prospective, cohort study with 6.574 patients diagnosed with COPD tested the hypothesis that the increased levels of inflammatory biomarkers in patients with stable COPD are related to the increased risk of exacerbation. The basal levels of CRP, fibrinogen and WBC were measured in patients with stable COPD. The results of this study showed that concurrently elevated CRP, fibrinogen and WBC levels in COPD patients are linked to exacerbation risk, even among individuals with a mild form of COPD and those without previous exacerbations (5). Two large studies demonstrated that increased CRP, fibrinogen and WBC are related to a double to four-fold increased risk of comorbidities in COPD; these biomarkers may also help clinicians in the stratification of COPD comorbidities (6). However, the above-mentioned studies examined the role of biomarkers in the settings of stable COPD; so far, little has been done to explore the relationship between inflammatory biomarkers and CD in AECOPD patients. In our study, univariate analysis showed that CRP, fibrinogen and NT-proBNP are all associated with increased hospital mortality, although this significance was lost after adjusting for low EF.

CD are one of the most common comorbidities in COPD patients and one of the major causes of death in AECOPD. Endothelial dysfunction and accelerated atherosclerosis due to inflammatory processes increase the incidence of CD in COPD. It has been shown that arterial hypertension is present in over 50% and chronic HF in more than 20% of COPD patients. Even a small reduction in FEV1 (less than 10%) doubles the myocardial infarction risk (10). In our study, 24.2% patients had LVSD (defined as LVEF <50%), arterial hypertension was present in 77.8% and coronary artery disease in 14.9% of all patients. Other common comorbidities were diabetes (19.6%) and malignant disease (3.6%). Except for LVSD, we observed no association between the presence of comorbidities and hospital mortality. Hospital mortality in the group of subjects with concomitant LVSD was 19.2% and was higher than that in subjects who had preserved LVEF (4.8%). When adjusted for levels of CRP, patients with EF <50% had a 3-fold increased risk of hospital death. In the study of 1.016 hospitalised COPD patients, Connors et al. (11) observed a hospital mortality rate of 11%. This is similar to our study, but the risk of death among our patients was markedly influenced by the presence of LVSD. Soler-Cataluña et al. (12) clearly showed that the outcome of COPD hospitalised patients in terms of survival is related to the frequency and severity of exacerbations. Among the most common causes of mortality in COPD are coronary heart disease and congestive HF. These diseases are the cause of death more often than respiratory failure due to the AECOPD itself (13,14).

When assessing mortality in AECOPD patients, it is important to take into account the presence of right HF. Chronic cor pulmonale is a well-known cause of death in COPD. The aetiopathogenesis of cor pulmonale in COPD patients is complex and includes several mechanism such as hypoxaemia, polycythaemia, inflammation and endothelial dysfunction that promote pulmonary hypertension (15). In our study, cor pulmonale was present in 34.4% of patients. Chronic core pulmonale was more common compared to LVSD but did not influence the mortality, probably because all of our patients had severe COPD and a significant number presented with chronic cor pulmonale.

The presence of pro-inflammatory state in COPD is also closely related to cardiovascular function. De Gennaro et al. (16) examined the association between systemic inflammatory response syndrome in 31 subjects with non-ischaemic dilated cardiomyopathy (NIDC). Results showed that LVEF was inversely correlated to levels of CRP and fibrinogen. The correlation between HF and inflammatory markers remained statistically significant after adjustment for age, sex and cardiovascular risk factors. In NIDC, increased values of inflammatory markers were in proportion to the severity of symptoms and impairment of systolic function. It has also been shown that systemic inflammation might be related to deterioration of New York Heart Association class (16). In our study, we observed a mild negative correlation between CRP and EF, while the NT-proBNP and CRP correlated moderately. In the Brazilian study, conducted with the aim of examining the prognostic significance of CRP in subjects hospitalised for HF, patients with CRP values over 3 mg/dL had an increased mortality. In a multivariate analysis, CRP was found to be the most important independent prognostic factor (17).

Both frequent exacerbations and the presence of coronary heart disease are important predictors of mortality in COPD patients. In addition, both of these are related to an increased inflammatory state that could be easily measured by routine laboratory tests. Our study demonstrated that AECOPD patients often present with LVSD, and that the latter is related to a higher level of inflammatory biomarkers. One of the strengths of our study was that all patients underwent a TTE, which is not routinely done in AECOPD, making it difficult to sometimes recognise which patients have impaired EF, especially in the absence of clinical manifestations of HF. As these patients are at an increased risk of hospital death, more detailed cardiovascular examination should be considered in patients who present with a higher level of inflammation.

This study has several limitations. We did not assess the presence of bacterial colonisation of the airways or the existence of bronchiectasis, which may also cause a higher burden of inflammation in patients with COPD. The presence of bacterial colonisation is difficult to determine in AECOPD as this would warrant specifically designed studies of patients in which the presence of airway colonisation had been previously evaluated. Similarly, the effect of bronchiectasis on inflammatory markers would require high resolution chest tomography, which is not routinely performed in AECOPD. Finally, the examination of mortality risk factors is limited due to a relatively low number of non-survivors. In addition, since this was an exploratory study, future studies are warranted to confirm the observed relationship and elucidate the complex relationship between level of inflammation, exacerbation and cardiovascular comorbidities.

In conclusion, our study demonstrated that one quarter of patients hospitalised for AECOPD present with LVSD. Patients with EF <50% present with higher levels of CRP and fibrinogen and have a 3-fold increased risk for hospital mortality that is independent of age and levels of hypoxaemia and pro-inflammatory biomarkers.

## Figures and Tables

**Table 1:**
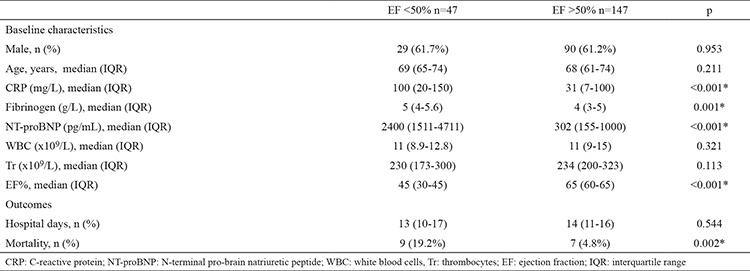
The differences in baseline characteristics and outcomes between chronic obstructive pulmonary disease patients with and without left ventricular systolic

**Table 2:**
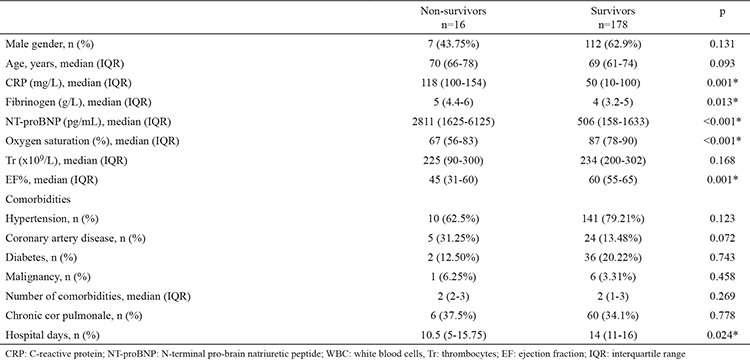
Risk factors for mortality in severe chronic obstructive pulmonary disease patients admitted for acute exacerbation chronic obstructive pulmonary disease

**Table 3:**
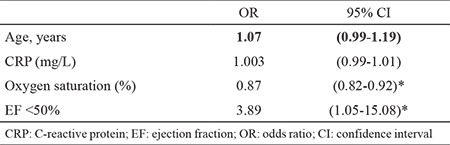
Multivariate analysis of factors associated with hospital mortality
